# Perioperative measurement of urinary oxygen tension as a tool in the prevention of acute kidney injury?

**DOI:** 10.1186/cc13568

**Published:** 2014-03-17

**Authors:** L Desteghe, W Boer, Q Swennen, V Pennemans, M Vander Laenen, K Engelen, C De Deyne, F Jans

**Affiliations:** 1 Hasselt University, Diepenbeek, Belgium; 2 Ziekenhuis Oost-Limburg, Genk, Belgium; 3 Biomedical Research Institute, Diepenbeek, Belgium

## Introduction

Acute kidney injury (AKI) remains a common complication after cardiopulmonary bypass (CPB) and can be diagnosed by serum creatinine [1]. However, serum creatinine is an insensitive and nonspecific biomarker [2]. This study was designed to investigate whether a correlation exists between urinary oxygen tension (UOT) and early markers of AKI. The aim was to evaluate whether UOT could provide warning signs of an insufficient renal oxygen supply, which can lead to postoperative AKI.

## Methods

Fourteen subjects undergoing cardiac surgery with CPB were included in this prospective clinical pilot study. UOT was measured perioperatively in all patients, both in the operating room (before, during and after CPB) and in the ICU. Biomarkers of AKI in blood and urine were measured preoperatively and postoperatively at 3, 6, 12 and 24 hours after the initiation of CPB. These included serum creatinine and the early urinary biomarkers kidney injury molecule-1 (KIM-1), neutrophil gelatinase-associated lipocalin (NGAL) and cystatin C. Student's *t *tests and Mann-Whitney tests were used to compare continuous variables.

## Results

There was a significant decrease in UOT between the start of CPB (138.44 ± 22.19 mmHg) and the lowest UOT during CPB (107.70 ± 23.28 mmHg) (*P *= 0.001). Dividing the subjects into two groups according to the Acute Kidney Injury Network (AKIN) classification, no significant differences were found in mean UOTs between the group of patients with a normal kidney function (*n *= 7) and the group with AKIN stage 1 or 2 (*n *= 7). For KIM-1, a significant difference between the two groups was found at 3 hours (*P *= 0.041) after the initiation of CPB. Further, for NGAL a significant increase in biomarker concentrations compared with the preoperative value was observed in the group with an AKIN stage 1 or 2 at all different postoperative time points (3 hours (*P *= 0.013), 6 hours (*P *= 0.003), 12 hours (*P *= 0.009) and 24 hours (*P *= 0.003)). On the contrary, there were no significant increased urinary NGAL levels measured in the group with the normal kidney function.

## Conclusion

This pilot study was not able to demonstrate any association between perioperatively measured UOT and markers of postoperative AKI. Additional laboratory and clinical studies will be necessary to further define the relationship between the UOT and new biomarkers of AKI.
